# The Relationship between Complements and Age-Related Macular Degeneration and Its Pathogenesis

**DOI:** 10.1155/2024/6416773

**Published:** 2024-01-02

**Authors:** Liyuan Chu, Chaoran Bi, Caiming Wang, Hongyan Zhou

**Affiliations:** ^1^Department of Ophthalmology, China–Japan Union Hospital of Jilin University, Changchun, China; ^2^College of Traditional Chinese Medicine, Hainan Medical University, Haikou, Hainan, China

## Abstract

Age-related macular degeneration is a retinal disease that causes permanent loss of central vision in people over the age of 65. Its pathogenesis may be related to mitochondrial dysfunction, inflammation, apoptosis, autophagy, complement, intestinal flora, and lipid disorders. In addition, the patient's genes, age, gender, cardiovascular disease, unhealthy diet, and living habits may also be risk factors for this disease. Complement proteins are widely distributed in serum and tissue fluid. In the early 21st century, a connection was found between the complement cascade and age-related macular degeneration. However, little is known about the effect of complement factors on the pathogenesis of age-related macular degeneration. This article reviews the factors associated with age-related macular degeneration, the relationship between each factor and complement, the related functions, and variants and provides new ideas for the treatment of this disease.

## 1. Introduction

**Figure 1:**
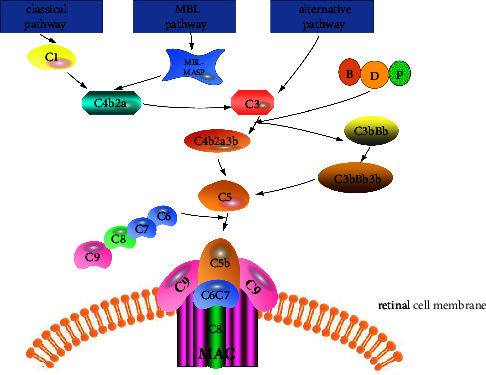
The classical pathway and the MBL pathway activate C1 and MBL-MASP-2, respectively, cleaving C2 and C4 to form C3 convertase. C4b2a combines with C3b formed after C3 cleavage to produce C5 convertase. In the alternative pathway, factor B combines with C3b, which is spontaneously hydrolyzed by C3 under the action of factors D and P to generate C3 convertase and C5 convertase. Both of the above enzymes can activate and cleave C5 and participate in the formation of the subsequent MAC.

Research shows that the number of people with age-related macular degeneration (AMD) is expected to reach nearly 300 million by 2040 [1]. Patients with early onset of AMD have no obvious symptoms, and fundus examination can show macular pigment disorders. According to the clinical manifestations, the disease can be divided into two categories, i.e., wet and dry, and the dry nature accounts for about 80%∼90% in the clinic. The course of dry AMD is slow, with a gradual and irreversible decrease in central visual acuity. The pathological manifestations include atrophy and dryness of the retinal pigment epithelium (RPE) layer. Effective treatment for this disease is currently lacking [2]. Patients with wet AMD usually have reduced or even loss of central visual acuity for several weeks or months, and the pathological manifestations are mainly neovascular. This neovascularization can develop subretinal through the choroid and is fragile, easily leaking serous fluid in the blood vessels to the surrounding tissues, causing an inflammatory response in the surrounding tissues [3]. At present, anti-VEGF therapy is being investigated as a treatment modality [4]. The complement system consists of more than 50 proteins, some of which are capable of recognizing pathogens in the fluid phase. The complement system reacts to environmental changes through three major pathways: the classical pathway, the alternative pathway, and the lectin pathway (or MBL pathway) [5]. The classical pathway and the MBL pathway activate C1 and MBL-MASP-2, respectively, to cleave C2 and C4 to form C3 convertase. C3b formed after C3 cleavage combines with C4b2a to produce C5 convertase. In the alternative pathway, factor B combines with C3b, which is spontaneously hydrolyzed by C3, with the assistance of factors D and P to generate C3 convertase and C5 convertase. Both of the above two enzymes can activate and cleave C5 and participate in the formation of the subsequent membrane attack complex (MAC) [6]. MAC can form a transmembrane channel on the cell membrane, allowing water and small soluble molecules to enter the cell through the channel. This causes cell rupture and chemokine release, resulting in an inflammatory response [7] (Figure 1). During the pathogenesis of AMD, the complement pathway is activated, and the product MAC can directly destroy retinal cells and gradually accumulate in the basal layer of retinal pigment epithelial cells to form drusen. Aggregation between Bruch's membrane and the retinal pigment epithelium results in retinal pigment epithelium-Bruch's membrane-choriocapillary complex degeneration. If the lesion reaches the retinal pigment epithelium and the neuroepithelial layer, it can lead to the formation of subretinal neovascularization and severely damage the patient's vision [8]. However, a significant correlation has been found between complement protein levels and age, BMI, and so on, which are also factors that accelerate the incidence of AMD [4]. At the same time, the coding variation of complements can also affect retinal function by changing the serum complement levels [9]. This article summarizes the 6 pathogenesis-related factors, their relationship with complements, and the complement function and mutants related to the pathogenesis of AMD.

## 2. The Pathogenesis of AMD

### 2.1. Mitochondrial Dysfunction and Oxidative Stress

The retina is one of the highest oxygen- and energy-consuming tissues in the human body. Mitochondria are double-membrane organelles and are the primary source of energy in cells; mitochondrial dysfunction has a significant impact on the retina. Under normal circumstances, the mitochondrial outer membrane regulates the entry and exit of ions and proteins into and out of cells through various channels and transporters. The inner membrane of the cell has a large surface area. Through the formation of ridges, various enzymes involved in electron transfer, tricarboxylic acid cycle, oxidative phosphorylation, and other processes are carried, providing energy for cells [10]. A part of the oxygen in the inner membrane is not completely reduced to form ROS during oxidative phosphorylation. ROS can regulate the communication transcription factor between mitochondria and the nucleus to transmit information [11]. ROS increases during the pathogenesis of AMD. Many photoreceptors exist in the retina, especially in the macular region. The photoreceptors are rich in unsaturated fatty acids and are targets of lipid oxidation [12]. In addition, there are many photosensitizers in RPE and photoreceptors (rhodopsin and lipofuscin) [13]. The photosensitizers undergo a photochemical reaction in response to the light perceived by the visual cycle, but this photochemical reaction results in the production of ROS [14]. Due to the high oxygen consumption and high concentration of unsaturated fatty acids and photosensitizers in the retina, the organ is prone to oxidation and antioxidation imbalance [15]. Oxidative stress seems to play a pivotal role in the pathogenesis of AMD, with a significant increase in ROS levels [16]. Excessive ROS impairs protein and lipid metabolism and damages mitochondrial DNA (mtDNA) in the matrix. mtDNA is a 16 kb circular double-stranded DNA without introns. 98% of the genome controls the expression of functional proteins such as 16sRNA and 12sRNA, and 2% controls its own replication, which is located in the d-loop. However, mtDNA lacks proofreading and repair functions, and under normal circumstances, mitochondrial transcription factor A (TFAM) and the nucleoid complex of various proteins are protected from abnormal external interference. Sustained ROS production damages mtDNA beyond repair capacity [17]. Therefore, most of the molecules involved in cellular oxidative phosphorylation (OXPHOS) are blocked due to mtDNA damage. This leads to the dysfunction of the complex electron transport chain in the inner mitochondrial membrane that generates ROS, which further stimulates the production of ROS [18]. Moreover, ROS production preferentially damages mtDNA [19], and mtDNA damage in the macula is more severe than in other retinal pigment epithelium regions [20]. Damaged mitochondria open their own permeability transition pores, releasing internal mitochondrial proteins and mtDNA, etc. [21]. These mtDNA and mitochondrial proteins disrupt the balance of cellular oxidative stress, promote the formation of apoptotic bodies, and promote inflammation [22]. In addition, the retina is more sensitive to light with shorter wavelengths. After long-term photodamage, the extraphotoreceptor segment (POS) with rich content of polyunsaturated fatty acids (PUFA), the intermediate produced by internalization and degradation of CD36 and MerTK, is ingested by lipofuscin particles or other photosensitizers in the retina, and lipofuscin can directly interact with light to produce ROS [23]. It can also be combined with N-retinoate-N-retinoate ethanolamine (A2E), that can cause fundus autofluorescence, to produce ROS. At the same time, the photoreceptors are continuously stimulated, which can accelerate the renewal rate of the outer segment, and produce more and more ROS, causing damage to the retina. Retinal RPE cells can engulf POS and cause respiratory bursts, known as ROS bursts [24].

### 2.2. Inflammation

The blood-retinal barriers (BRBs) prevent immune molecules from entering the omentum parenchyma, and resident immune cells, such as microglia and the complement system, contribute to immune privilege. Therefore, the immune privilege of the retina is involved in the retinal damage response [25]. Microglia are mainly distributed in the inner layer of retinal nerves and are regulated by CX3CL1/CX3CR1, C2, and other molecules. They can be divided into a proinflammatory phenotype (M1 type) and an anti-inflammatory phenotype (M2 type). After stimulation, M1-type microglia can change into an amoeba shape and migrate to the extraretinal and subretinal areas to phagocytose pathogens, receptors, and damaged cell debris [26]. The release of inflammatory factors such as TNF-*α* and IL-1*β* induces inflammation [27]. The M2 type is the opposite of the M1 type, and its release of IL-10, TGF-*β*, VEGF, and so on counters inflammation and promotes cell repair [28]. In the retina of AMD patients, inflammatory pathways are activated, IL-1*β* increases, and inflammasomes are formed [29]. Studies have shown that IL-1*β* stimulates the expression of chemokines such as Ccl2 in Muller cells and retinal RPE cells and promotes the accumulation of macrophages. The types of macrophages are similar to those of microglia, with the antivascular M1 type and the provascular M2 type exerting opposite effects, regulating the growth of retinal and choroidal blood vessels [30]. Inflammasomes are multiprotein complexes composed of sensor proteins, adaptor proteins, and proenzymes. It is a class of oligomeric complexes that act as receptors to recognize microorganisms and cell products. In the retina, inflammasome assembles with procaspase-1 after retinal activation, after which it is assembled to divide and dissolve into caspase-1. Caspase-1 cleaves some propeptides (e.g., IL-18 and IL-1*β*) into active forms to activate inflammation [31]. They accelerate Bruch's membrane degradation and choroidal neovascular degeneration [32]. Studies have shown that the activation of inflammasomes promotes retinal neovascularization in AMD patients, and the targets are mainly located in non-RPE cells [33]. C-reactive protein can be found in drusen in the choroid basal layer, where it acts as a regulator of activated platelets and monocytes, participates in various inflammatory pathways, and targets choroidal cells to destroy [34].

### 2.3. Apoptosis

Apoptosis is a programmed cell death process characterized by the formation of membrane vesicles, cell shrinkage, nuclear fragmentation, and apoptotic bodies [35]. It is a programmed death that begins in utero and is opposed to mitosis and can occur through both intrinsic and extrinsic pathways. (1) The intrinsic pathway, also known as the mitochondrial pathway, is usually activated by increased ROS, lack of oxygen, protein or DNA damage, and so on, which can stimulate mitochondria to accelerate the production of cytochrome C and then activate caspase for subsequent reactions. At the same time, the intrinsic pathway is regulated by the Bcl-2 family and fluctuates under the regulation of proapoptotic factors (such as Bax, Bak, Bad, and Bcl-x) and antiapoptotic factors (such as Bcl-2 and Bcl-xl). (2) External pathways can occur by the death receptor protein family, such as Fas binding to Apo1 and TNFR1 receptor binding to tumor necrosis factor (TNF), and then activating caspase [36]. Dysfunctional mitochondria and endoplasmic reticulum are detected in the retina of AMD patients with visual impairment [37]. The endoplasmic reticulum is a tubular network mainly responsible for protein synthesis, folding, and transport. The stress response to oxidative damage leads to protein maturation disorders and induces the activation of caspase-4 [38]. Apoptosis is regulated by the caspase protein family, and activated caspase-4 activates downstream caspase-3/6/7 along with the signaling pathway, triggering apoptosis in target cells [39]. Moreover, from the perspective of toxicology, cell apoptosis shares some common inducing factors with AMD pathogenesis [40]. Caspase and cytochrome c release following mitochondrial damage [41], and the production of IL-1*β*, IFN-*γ*, IL-6, TNF-*α*, and oxLDL by macrophages after phagocytosis of pathogens can induce apoptosis [42]. Furthermore, A2E (N-retinyl-N-retinylidene ethanolamine) can generate a large amount of ROS and can also cause apoptosis [43]. These are all important factors in the pathogenesis of AMD, highlighting the close relationship of apoptosis to AMD.

### 2.4. Gut Microbiota

There are about 100 trillion microorganisms in the human gut. Due to people's different lifestyles, body mass index, and cultural and dietary habits, the microbiota in the human gut vary considerably [44]. The disturbance of intestinal flora may damage the intestinal vascular barrier and increase the permeability of the intestinal tract. Therefore, the stability of the intestinal flora is essential for maintaining the health of the body [45]. In recent years, studies have found that the impact of gut microbiota energy metabolism, signal transmission, and barrier regulation on the body is not limited to the intestinal lumen but also involves the central nervous system [46]. The retina is an extension of the central nervous system, and the concept of the “microbiota-gut-retina axis” is being researched [47]. Aging is an unavoidable cause of AMD. Studies have shown that with the natural aging process, the types and quantities of microbiota in the gut also change [48]. Furthermore, a high-sugar and high-fat diet is also related to changes in gut flora and the pathogenesis of AMD [49]. In a mouse model of AMD, a larger proportion of *Clostridium* species were found in the intestine of mice on a high-fat diet, while a low-fat diet promoted the growth of the S24-7 family and Bacteroidetes. *Clostridium* species increase the systemic inflammatory response and promote choroidal neovascularization, while Bacteroidetes inhibit the pathogenesis of AMD in the body [50]. Firmicutes were more prevalent in AMD patients than in controls, while Bacteroidetes were less abundant. Lower levels of bacteria linked to fatty acid elongation were found in patients with AMD [51]. In contrast, *Escherichia coli* and *Helicobacter pylori* in the intestinal lumen lead to increased ROS levels [52].

### 2.5. Lipid Metabolism

Drusen is an early clinical feature of AMD, which is characterized by a localized dome-shaped basal linear deposit formed between the inner collagen layer of Bruch's membrane and the RPE substrate [53]. About 40% of these sediment components are lipids such as cholesterol, triglycerides, fatty acids, and apolipoproteins [54]. Therefore, drusen is also referred to as “oil leak on the Bruch membrane” [55]. Cholesterol is a lipid component that performs important functions in living organisms and can be synthesized by nucleated cells. It is an essential component of cell membranes, steroids, and neuronal synapses and is part of the transcriptional regulation of genes [56]. Due to the high metabolic characteristics of RPE cells, in order to avoid the accumulation of cholesterol in the cell, RPE cells can expel excess cholesterol outside the cell through the subretinal space and choroidal blood vessels; otherwise, this will affect the transduction process of light in the retina. Among them, the efflux mechanism of ABCA1/ABCG1 is mentioned more; if cholesterol accumulation occurs in RPE cells, ATP-binding box transporters A1 (ABCA1) and G1 are activated, cholesterol is flipped to the cell surface through the inner lobule, and cholesterol that is flipped to the cell surface plays a role with apoliporeceptor protein to play subsequent biological functions [57]. Moreover, these lipids are easily oxidized to advanced glycation end products, such as carboxyethylpyrrole, malondialdehyde, and 4-hydroxynonenal [58]. These products promote the progression of AMD by accelerating macrophage aggregation, cytokine release, and neovascularization [59]. Studies have shown that high-density cholesterol (HDL-C), serum triglycerides (TG), and low-density cholesterol (LDL-C) are associated with early AMD [60]. In symptomatic AMD patients, improvements in eyesight and adverse symptoms have been observed after statin treatment [61]. ApoE is a popular apolipoprotein derived from systemic circulation and RPE cells and has recently been linked to this disease. ApoE packages cholesterol and fat and transports them via the circulation to the retina, where cholesterol and fat are released to ensure retinal energy supply [62]. Studies have reported a positive correlation between ApoE2 polymorphism and the occurrence of AMD, while ApoE 4 polymorphism is negatively correlated with the occurrence of AMD. Furthermore, glucose metabolism and lipid metabolism disorders were found in mice lacking ApoE, and substances similar to basal linear deposits were seen in retinal RPE and Bruch's membrane [63]. Therefore, lipid disorders and AMD pathogenesis are related to each other.

### 2.6. Autophagy

Eukaryotes can maintain gene conservation through the autophagy pathway. Under normal circumstances, autophagy can degrade aging and damaged intracellular substances through organelles such as lysosomes to maintain intracellular turnover and circulation stability. Its pathways can be divided into macroautophagy, microautophagy, and chaperone-modified autophagy, which are regulated by AMP-activated protein kinase (AMPK) and the mammalian target of rapamycin (mTOR) pathways [64]. Macroautophagy is the main autophagy pathway of cells; when the cell is misfolded and pathogens invade, it can be wrapped by the double-membrane structure to form autophagosomes, and after autophagies, lysosomes fuse to form autophagic lysosomes and finally degrade the substrate. Microautophagy is degraded by lysosomal membrane invagination, wrapping the degraded substrate into the lysosome. Chaperone protein-mediated autophagy is common in mammals, and this autophagy mainly relies on lysosome-related transporters (such as lysosome-associated membrane protein 2A and heat shock protein) to transport substrates to lysosomes for degradation [65]. Retinal RPE proteolysis is impaired during AMD, and the accumulated proteins after chronic oxidative stress are deposited as lipofuscin in RPE, which promotes the formation of drusen [66]. The main component of lipofuscin is A2E, which inhibits autophagy and aggravates RPE cell damage [67]. In the early stage of the disease, RPE cells can remove the accumulated waste through autophagy, but as the disease progresses, the lysosomal activity in the cells decreases, and the RPE cells become disordered [68]. Decreased lysosomal activity and elevated autophagy markers ATG5, LC3, and so on have been observed in AMD patients. In the autophagosome formation process, the ratio of soluble LC3-I and lipid-bound LC3-II can be used as a standard to evaluate autophagosome formation [69]. The model group mice showed retinal RPE hyperplasia, pigmentation disorder, and accumulated oxidized protein substances after silencing the core genes ATG5 and ATG7 of mouse autophagy. The content of misfolded proteins in AMD far exceeds the repair capacity of heat shock proteins. Misfolded proteins tend to form harmful aggregates. After being encapsulated, fusion with lysosomes causes cellular inflammatory responses and autophagy [70]. Autophagy also affects POS in RPE cells, and since each RPE cell needs to assist multiple rods, a large amount of POS is required to be constantly updated. During the renewal process, autophagies start at the apex of POS and degrade the disk structure of POS. In addition, a large number of studies have shown that the onset of AMD can cause excessive autophagy in RPE cells, the number of autophagies in RPE cells is increased compared with that in the control group, and autophagy-related proteins and autophagy flow are reduced. Inhibition of RPE autophagy protects against photoreceptor damage in RPE cells [71].

## 3. The Pathogenesis of AMD and Its Relationship with Complement

### 3.1. Complement and Mitochondrial Dysfunction

CD46 and C1q are complements that mainly affect mitochondrial dysfunction. Some studies have found that mitochondria-related genes have been altered by completely suppressing the complement system of experimental animals with fatty liver models [72]. Therefore, it is speculated that complements play a role in mitochondrial function [73]. It was subsequently found that C1q internalized by CD8+ T cells can bind to the C1q receptor on the mitochondrial membrane and regulate ATP synthase complex formation. The ATP synthase complex functions to maintain mitochondrial morphology and mitochondrial membrane potential [17]. At the same time, it was found that CD46 is a protein receptor present on the membrane of nucleated cells for complement regulation of cells. When the body is at rest, it exists in the form of CD46-CYT-2. When the body is stimulated, it binds to C3b in the form of CD46-CYT-1 and enters cells. CD46 entering cells participates in the expression of genes related to glucose transporter 1 and L-type amino acid transporter 1, promoting the utilization of glucose and amino acids by cells, and maintaining the level of mitochondrial metabolism [74]. The reduction of CD46 on the surface of RPE cells in AMD patients also affects the subsequent mitochondrial production capacity [75]. Furthermore, MBL can localize C4 to mitochondria to activate the complement pathway. The identified mitochondria activate their stress protein (mortalin), originally located in mitochondria, which relocates to the cell membrane and activates the release of complement [76]. In vitro, cultured renal tubular epithelial cells were added with C5a, and decreased mitochondrial oxidative respiration capacity and increased ROS production were reported [77]. This may be related to the increase of Ca^2+^ uptake after the activation of corresponding C5a on the mitochondria and the increase of its internal Ca^2+^ concentration [78].

### 3.2. Complement and Inflammation

Complement C3 is a major proinflammatory protein. The complement pathway is activated in the pathogenesis of AMD. Elevated levels of C3a, C5a, and MAC directly activate the NF-*κ*B pathway, stimulating the secretion of interleukin from monocytes and the secretion of trypsin and chymase from mast cells [79]. Complements also induce ATP efflux and initiate the “complement-metabolism-inflammasome” signaling axis to promote NLRP3 inflammasome formation [80]. In addition, NF-*κ*B promotes inflammatory and oxidative responses [81]. Chymase can hydrolyze C3 to C3a, further intensifying the activation of the complement pathway [82], and the NLRP3 inflammasome can be activated by macrophages that have phagocytosed C1q. After activation, it catalyzes the cleavage of the caspase-1 precursor, leading to the activation of inflammatory factors IL-18 and IL-1b [83]. The alternative complement pathway induces the release of extracellular and intracellular damage-associated molecular pattern (DAMP) molecules, which promote the expression of inflammatory factors in RPE cells [84]. Under the action of C3b and C5a, C3a promotes the respiratory burst of neutrophils and exacerbates the production of ROS [85]. The chemotaxis and phagocytic functions of neutrophils are inhibited under extended exposure to higher concentrations of C5a [86]. In an AMD experimental model, increased levels of IL-6, IL-8, and GM-CSF were detected after the complement factor H gene was silenced [87]. At the same time, complement factor-related protein 1 can induce NLRP3 production through C-terminal binding to G protein-coupled receptors [88].

### 3.3. Complement and Apoptosis

C3 and C5 are complements that mainly affect apoptosis. Studies have shown that C3a delays the decline of proapoptotic cells, such as CD4+ T cells and macrophages. In the absence of C3a, T cells fail to differentiate into IFN-*γ*-producing Th1 effector cells [89]. IFN-*γ* promotes the apoptosis of RPE cells [90]. Similarly, increasing the concentration of C3a in the culture environment of macrophages led to a significant reduction in the apoptosis rate of macrophages, resulting in the prolonged release of IL-1*β*, TNF-*α*, prostaglandins, and other molecules [91]. Experiments have shown that TNF-*α* is an external factor that promotes apoptosis [92]. The combination of C5a and C5a receptors activates the NF-*κ*B pathway to regulate the cell cycle, where C5a causes retinal cell cycle arrest at the G1 phase. The C5a receptor promotes the replication of cell genetic material and allows the cell cycle to progress to the G2/M phase [93]. C5a directly acts on vascular endothelial cells and increases their permeability. C5a combines with its receptor and attracts neutrophils, eosinophils, monocytes, and chemotaxis to the damaged site [94]. Furthermore, C5a receptors can activate the antiapoptotic factor Bcl-2 to inhibit apoptosis [37]. C1q can bind to the corresponding receptor, reducing cell viability and promoting the expression of the tumor suppressor gene p53. Therefore, apoptosis is regulated by the activity of the Bcl-2 family and mitochondrial function [95].

### 3.4. Complement and Gut Microbiota

Factors C3 and D are complements that mainly affect apoptosis. The gut is one of the most abundant microbial communities in the human body, and these colonies play an important role in regulating functions such as immunity [96]. In order to explore the relationship between complements and intestinal flora, some studies have found an increase in *Escherichia coli* in the intestinal tract after silencing the CFD gene. The increased *E. coli* not only affects distant target organs through the “microbiota-gut-retina axis” but also stimulates the macrophages in the intestine to cause digestive system diseases such as colitis [97]. The fecal microorganisms in the colon of 16-week-old mice with C3 gene knockout were analyzed, revealing decreased anaerobic bacteria and kinetobacteria in the experimental group lacking C3 compared to the control group; in contrast, the flora of fungi and Bacteroidetes increased compared with the control group [98]. Firmicutes and Bacteroidetes account for about 75% of the adult intestinal flora, which play a role in protecting the structure and metabolism of intestinal epithelial cells [99]. *Yersinia pseudotuberculosis* and *Akkermansia muciniphila* were incubated in patient serum. The experiment revealed that C4b could be involved in the intestinal defense response, which was positively correlated to the degree of inflammation [100]. CFH and its polymorphism-produced mutants (rs10490924) may also have an impact on the distribution of the intestinal flora [101].

### 3.5. Complement and Lipid Metabolism

Many complements, such as CFH, CFD, C1, C3, and C7–9, are related to lipid metabolism. Studies have shown that apolipoprotein E can bind to domains 5–7 of CFH and that apolipoprotein J can bind to C7, C8, and C9 to affect the complement activation pathway. Lipoprotein accumulation is seen in RPE when mutants are formed due to the presence of SNP sites in the complement gene [102]. Similarly, ApoE isoforms can form C1q-ApoE complexes with C1q, causing leukocytes to infiltrate the choroid and also activate the complement cascade. Studies have shown that overexpression of CFB in adipocytes promotes the differentiation and maturation of adipocytes. Furthermore, CFB also prompts adipocytes to produce more enzymes related to lipid synthesis, such as acetyl-CoA carboxylase, thereby increasing lipid levels [59]. The serum level of CFD in mice increased after a high-fat diet, and CFD can reduce the expression of inflammatory factors and the absorption and de novo synthesis of fatty acids in the liver [103]. C3 is involved in the metabolism of triglycerides in adipocytes, and the level of C3 in serum is proportional to the level of low-density lipoprotein cholesterol. At the same time, C3 is also regulated by chylomicrons that carry transthyretin [104]. The lipid content in hepatocytes of mice with C5 gene knockout is higher than that of controls with activated C5 [105]. In addition to affecting the complement system, lipids can also affect related complement regulatory proteins. For example, CD59 relies on cholesterol localization for subsequent processes, and excess cholesterol leads to the accumulation of MAC [106].

### 3.6. Complements and Autophagy

C3, C5, and CD46 are complement proteins that mainly affect autophagy. The experiment revealed that the mice with C3a and C5a receptor knockout had obvious mitochondrial autophagy and had decreased antihost disease ability than the control group. Interestingly, some C3 exists in the cytoplasm, and the autophagy in the cytoplasm that C3 participates in cannot be replaced by extracellular C3 [107]. In podocytes cultured in vitro, it was found that MAC can promote the conversion between LC3-I/II and can also increase the levels of autophagy-related markers such as p62 and Beclin1 [108]. In general, complements also indirectly regulate autophagy through related regulatory proteins and corresponding receptors on the cell surface. Among them, CD46 is a cell surface transmembrane protein with two transduction cell membrane signal structures (Cyt-1 and Cyt-2), which hydrolyze C3b and C4b with the help of CFI to induce autophagy in nucleated cells [109]. V-set and immunoglobulin domain-containing 4 (VSIG4) itself aggregate autophagy ubiquitin-binding receptor proteins such as p62 to induce autophagy and can also bind to C3b and iC3b to regulate subsequent autophagy [110].

## 4. General Situation of Several Common AMD-Associated Complement Factors and SNPs

### 4.1. Complement Factor H (CFH)

#### 4.1.1. Function

CFH is a soluble protein synthesized by the liver and RPE cells. Studies have shown that CFH plasma levels in young, elderly, and AMD patients are about 233 mg/L, 269 mg/L, and 288 mg/L, respectively [111]. However, age, smoking, and other factors may lead to increased CFH levels [112]. Complementary control protein modules are encoded on the chromosome position 1q32 and contain highly conserved repeat units. CFH tightly connects RPE cells to each other and protects cells through an apoptotic program [113]. Its own complementary control protein module inhibits C3 convertase activity by electrostatic repulsion with factor B. Furthermore, CFH competes with factor B to bind C3b, inactivating C3b with the assistance of factor I, thus inhibiting the alternative complement pathway [114]. Klein et al. have reported the presence of CFH in Bruch's membrane in AMD patients [115]. Studies have shown that the complement factor H-related protein (CFHR) exerts opposite effects on CFH, strengthening the activation of C3b to activate the complement pathway. Increased levels of CFHR have been found in the plasma of advanced AMD patients [116]. In addition to CFH, five CFHR proteins (CFHR1∼5) form a structurally related protein family. However, the biological role of CFHR proteins remains unclear [117].

#### 4.1.2. SNPs

Complement factor H gene-level changes, such as single-nucleotide polymorphisms (SNPs), lead to changes in CFH levels and may result in disease. For example, mutation of the base thymine T to cytosine C in the complement factor H gene results in a corresponding change in translation of histidine to tyrosine, leading to the formation of rs1061170 [118]. Decreased binding to glycosaminoglycans on the cell membrane surface alleviates the inhibition of the alternative pathway and further damages the retina [87]. Another SNP site of CFH is prone to form rs800292, in which guanine G is mutated to adenine A, resulting in isoleucine being replaced by valine after translation. The protein secreted by rs800292 not only has decreased binding capacity to C3b but also inhibits its degradation [119]. The mutation frequency in the domestic population (43.3%) is slightly higher than that in the European population (40.8%). In contrast, the mutation frequency of rs1061170 in the domestic northern population is much higher than in the European population [120]. Studies have shown that the risk of developing AMD in people carrying both rs1410996 and rs1061170 is 15 times that of the normal population, but their effects are mutually independent [121]. Moreover, the SNP sites rsl1200638 (HTRA1 promoter) and rsl10490924 (the upstream 6.6 kb sequence of HTRA1) of the CFH gene can interact and are positively correlated with the pathogenesis of both types of AMD [122]. In addition, rs1061170 impairs the binding of H-like protein 1 and FH to Bruch's membrane, which can activate the complement pathway and accelerate the occurrence of AMD [123].

### 4.2. Complement Factors C1–9

#### 4.2.1. Function

Complement C promotes the occurrence of AMD. C3 is the most abundant, and its cleavage products, C3a and C3b, are also the hubs for the activation of the entire complement system [124]. C3a can stimulate inflammatory cells to release histamine, increase the permeability of the blood vessels in the retina, and produce edema; it can stimulate RPE cells to express VEGF and form new blood vessels [125]. C3a can also combine with autoantibodies to promote the production of collagens IV and VI, which are deposited under RPE [126]. In a previous study, C3 knockout mice developed earlier retinal degeneration after exposure to UV light [127]. Some studies have detected C3 RNA in the retina of patients, but no C3a receptor has been found, so it is speculated that the damage of C3a to the retina may occur through intercellular adhesion molecule-1 (ICAM-1) [128]. Activated C3 can cleave C4 into C4a and C4b, and C4a also promotes the release of histamine, but its activity is worse than that of C3a. C4b participates in the classical pathway of complement to form C3 and C5 convertases [129]. However, the relationship between C4 and AMD remains to be further studied. C5a is similar to C3a in some aspects, as it can also stimulate RPE cells to produce VEGF and can induce local inflammation through ICAM-1. In contrast, C5a receptors have been found on the surface of RPE cell membranes cultured in vitro [130].

#### 4.2.2. SNPs

C1 SNP rs2511989 and C2 SNP rs9332739 alleviate AMD [131]. rs2230199 (Arg80Gly) is formed by a mutation of the SNP site of C3 (most commonly, a base located in exon 3) from cytosine C to guanine G, leading to the posttranslational replacement of arginine to glycine. This mutation increases the risk of early AMD. Furthermore, the incidence ratio (2.6) of homozygous GG after mutation is roughly 1.5 times the incidence ratio of heterozygous CG (1.7) [132]. The knock-on effect between rs1047286 and rs2230199 results in the formation of another C3 variant, which could be related to AMD [133]. Grassmann et al. reported the correlation between C4 copy number variations (CNVs) and AMD [134].

### 4.3. Complement Factor B (CFB)

#### 4.3.1. Function

Factor B is a single-chain glycoprotein mainly secreted by hepatocytes, with a plasma concentration of about 200 *μ*g/mL. It is cleaved into Ba and Bb by activated factor D [135]. Bb can combine with C3b under the action of Mg^2+^ to form the polymer C3bBb, which is the rate-limiting step of the complement pathway [1]. The crystal structure of factor B contains the homologous repeat sequence of Ba in an antiparallel dimer, which may inhibit the binding of C3b to Bb, despite Ba not being directly involved in the process [136]. The study found that CFB can be expressed in the retina. AMD patients exhibit elevated CFB levels, which exert a stronger effect on drusen and Bruch's membrane compared to other layers [137]. CFB also causes RPE damage and promotes neovascularization [138] and can be detected in the aqueous humor of AMD patients with neovascularization [137].

#### 4.3.2. SNPs

The CFB gene is located on chromosome 6p21.3, and its SNPs rs4151667, rs641153, rs12614, and rs9332739 have decreased binding ability to C3b. This significantly reduces the incidence of AMD [139]. Some studies suggest that the CFB gene may be related to the C2 gene as the CFB gene and the C2 gene are only 500 bp apart on the same chromosome. However, the interaction between these two and their effect on the pathogenesis of AMD remains to be further studied [140].

### 4.4. Complement Factor D (CFD)

#### 4.4.1. Function

CFD is a serine protease, mainly produced by adipocytes and macrophages, which cleaves factor B in the alternative complement pathway and promotes the formation of C3bBb [141]. People with a higher body mass index (BMI) have relatively higher CFD content, which may be related to the amount of adipose tissue [142]. Elevated serum CFD levels have been observed in AMD patients. In addition, gender and age also impact the body fat content, and the CFD content varies accordingly. The normal range fluctuates between 1 and 2 *µ*g/mL [143]. When the body is in a healthy state, factor D is almost completely absorbed by the renal tubules after being filtered by the glomerulus. However, some diseases may cause increased levels of factor D in plasma and lead to nephron damage [144]. Factor D is produced in the form of a zymogen, and its maturation requires the excision of excess amino acids; this process is quick and may be completed during secretion, so factor D is generally found in its mature form [145]. However, this mature form is not in an active state. When factor B and C3b bind, factor D changes from a “locked” mature inactive state to a mature active state. When factor B and C3b are cleaved, factor D can be reabsorbed by the kidneys and utilized by the body [146]. A previous study exposed mice to constant light for 12 hours for 10 consecutive days, revealing that the CFD gene knockout mice had a lower prevalence of AMD and a lower degree of damage to retinal receptor cells than in the control group [147].

#### 4.4.2. SNPs

Some studies believed that CFD mutation could promote AMD progression [148]. rs1683564, rs35186399, rs1683563, rs3826945, and rs34337649 are the more common SNPs [149].

### 4.5. Complement Factor I (CFI)

#### 4.5.1. Function

CFI is a serine protease with a special structure and has poor activity in the free state [150], but it can cleave and inactivate C3b and C4b with the assistance of the complement activity regulator protein family [151]. It also accelerates the degradation of C3 convertase and C3bBb and inhibits the activation of the alternative complement pathway [8]. At the tissue level, CFI combines with *β*-amyloid in drusen, leading to retinal inflammation. At the same time, *β*-amyloid also inhibits the cleavage of C3b by CFI [152]. The incidence of AMD may be increased in patients with *β*-amyloid-related Alzheimer's disease. CFI activity increases with the progressive aggravation of AMD, along with its ability to degrade and passivate [153]. Recent studies have shown that a small amount of factor I can be detected in the serum of patients with advanced AMD [154]. However, since factor I is a normal component in plasma, elevated levels do not increase the risk of immunogenicity in the body [153]. Serum CRP (C-reactive protein) levels are also raised in AMD patients, which may be positively correlated with complement factor I [155].

#### 4.5.2. SNPs

rs141853578 is unfavorable for patients with advanced disease [156]. In contrast, rs13117504 allele G and rs10033900 allele C play a protective role in patients with advanced AMD [157]. rs2285714 allele T has a negative impact on advanced AMD patients [158]. However, a study investigated the mutant rs10033900 in a British population found no association between the mutant and AMD [156]. More than half of the CFI genetic variants are found in AMD patients, and lower serum CFI levels have been observed in these patients [159].

## 5. Conclusion

In view of the interaction between complements and AMD, it has been found that inhibiting complement activation can reduce the damage of RPE cells, thereby inhibiting the pathogenesis of AMD [106]. Therefore, many complement inhibitors were discovered. For example, POT-4 inhibits the conversion of C3 to C3a and C3b, whereas ARC1905 and eculizumab inhibit C5a. Ranibizumab is a factor D inhibitor, and CD59 inhibits MAC deposition on the retina [160]. Although complement inhibitors have entered the clinical research stage, their specific efficacy and safety may need further exploration. Recent clinical trials have shown that complement inhibitors APL-2 and Zimura have promising effects on geographic atrophy (GA). The combination of Zimura and ranibizumab has a favorable effect on vision in wet AMD patients [161]. However, the incidence of AMD and the mutation rate of related genes are also related to region and ethnicity, and the characteristics of the population in this region should also be considered [162].

**Figure 2:**
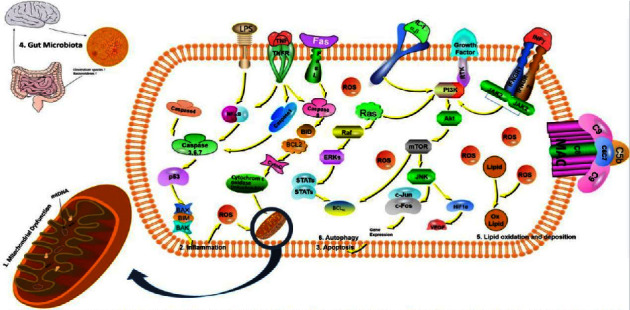
Changes in complement function accelerate the formation of MAC and activate PI3K, Fas, TNF, and other pathways. Excessive ROS and inflammatory factors are produced in retinal cells, causing cellular mitochondrial dysfunction, oxidative stress, lipid metabolism disorders, inflammatory response, apoptosis, and autophagy response. Studies have shown that changes in the structure of the intestinal flora can also exacerbate the formation of choroidal neovascularization. The combination of the above factors exceeds the compensatory capacity and results in AMD.

The pathogenesis of AMD is complex. Inflammation, apoptosis, autophagy, mitochondrial dysfunction, gut microbiota, and lipid disorders are involved, and complements are closely related to the occurrence of these factors. Complements contribute to the pathogenesis of AMD and potentiate other factors leading to AMD. The formation of MAC activates Fas, TNF, INF, and other pathways, which can regulate the cell cycle and promote apoptosis and autophagy. Activation of the caspase family of proteins affects the cell cycle and induces cellular inflammation. The production of ROS can damage mtDNA, leading to mitochondrial dysfunction. Intracellular lipids can also be oxidized, resulting in lipid accumulation. Moreover, elevated blood lipids also change the gut microbiota, acting on the retina through the “microbiota-gut-retina axis” to cause AMD (Figure 2). Therefore, studying the role of complement molecules in the occurrence and development of AMD can help clinicians formulate effective treatment plans for AMD patients. It will lay the foundation for AMD research in the future.

## Data Availability

No data were used to support the findings of this study.

## Abbreviations

AMD: Age-related macular degeneration
RPE: Retinal pigment epithelium
MAC: Membrane attack complex
CFH: Complement factor H
CFHR: Complement factor H-related protein
SNPs: Single-nucleotide polymorphisms
ICAM-1: Intercellular adhesion molecule-1
CNVs: Copy number variations
CFB: Complement factor B
CFD: Complement factor D
BMI: Body mass index
CFI: Complement factor I
CRP: C-reactive protein
mtDNA: Mitochondrial DNA
TFAM: Mitochondrial transcription factor A
OXPHOS: Oxidative phosphorylation
BRBs: Blood-retinal barriers
DAMPs: Intracellular damage-associated molecular patterns
A2E: N-retinyl-N-retinylidene ethanolamine
HDL-C: High-density cholesterol
TG: Triglycerides
LDL-C: Low-density cholesterol
AMPK: AMP-activated protein kinase
mTOR: Rapamycin
VSIG4: V-set and immunoglobulin domain-containing 4.

## References

[1] Mitchell P., Liew G., Gopinath B., Wong T. Y. (2018). Age-related macular degeneration. *The Lancet*.

[2] Sadda S. R., Guymer R., Holz F. G. (2018). Consensus definition for atrophy associated with age-related macular degeneration on OCT: classification of atrophy report 3. *Ophthalmology*.

[3] Grassmann F., Schoenberger P. G. A., Brandl C. (2014). A circulating microrna profile is associated with late-stage neovascular age-related macular degeneration. *PLoS One*.

[4] Acar I. E., Willems E., Kersten E. (2021). Semi-quantitative multiplex profiling of the complement system identifies associations of complement proteins with genetic variants and metabolites in age-related macular degeneration. *Journal of Personalized Medicine*.

[5] Hollyfield J. G. (2010). Age-related macular degeneration: the molecular link between oxidative damage, tissue-specific inflammation and outer retinal disease: the Proctor lecture. *Investigative Ophthalmology and Visual Science*.

[6] Merle N. S., Church S. E., Fremeaux-Bacchi V., Roumenina L. T. (2015). Complement system Part I-molecular mechanisms of activation and regulation. *Frontiers in Immunology*.

[7] Kumar-Singh R. (2019). The role of complement membrane attack complex in dry and wet AMD-from hypothesis to clinical trials. *Experimental Eye Research*.

[8] Cserhalmi M., Papp A., Brandus B., Uzonyi B., Józsi M. (2019). Regulation of regulators: role of the complement factor H-related proteins. *Seminars in Immunology*.

[9] de Jong S., de Breuk A., Bakker B. (2021). Functional analysis of variants in complement factor I identified in age-related macular degeneration and atypical hemolytic uremic syndrome. *Frontiers in Immunology*.

[10] Rahman J., Singh P., Merle N. S., Niyonzima N., Kemper C. (2021). Complement’s favourite organelle-Mitochondria?. *British Journal of Pharmacology*.

[11] Giorgi C., Marchi S., Simoes I. C. M. (2018). Mitochondria and reactive oxygen species in aging and age-related diseases. *International review of cell and molecular biology*.

[12] Jarrett S. G., Boulton M. E. (2012). Consequences of oxidative stress in age-related macular degeneration. *Molecular Aspects of Medicine*.

[13] Hunter J. J., Morgan J. I., Merigan W. H., Sliney D. H., Sparrow J. R., Williams D. R. (2012). The susceptibility of the retina to photochemical damage from visible light. *Progress in Retinal and Eye Research*.

[14] Beatty S., Koh H., Phil M., Henson D., Boulton M. (2000). The role of oxidative stress in the pathogenesis of age-related macular degeneration. *Survey of Ophthalmology*.

[15] Abokyi S., To C.-H., Lam T. T., Tse D. Y. (2020). Central role of oxidative stress in age-related macular degeneration: evidence from a review of the molecular mechanisms and animal models. *Oxidative Medicine and Cellular Longevity*.

[16] Kohen R., Nyska A. (2002). Invited review: oxidation of biological systems: oxidative stress phenomena, antioxidants, redox reactions, and methods for their quantification. *Toxicologic Pathology*.

[17] Fisher C. R., Ferrington D. A. (2018). Perspective on AMD pathobiology: a bioenergetic crisis in the RPE. *Investigative Opthalmology and Visual Science*.

[18] Liang F.-Q., Godley B. F. (2003). Oxidative stress-induced mitochondrial DNA damage in human retinal pigment epithelial cells: a possible mechanism for RPE aging and age-related macular degeneration. *Experimental Eye Research*.

[19] Ferrington D. A., Fisher C. R., Kowluru R. A. (2020). Mitochondrial defects drive degenerative retinal diseases. *Trends in Molecular Medicine*.

[20] Terluk M. R., Kapphahn R. J., Soukup L. M. (2015). Investigating mitochondria as a target for treating age-related macular degeneration. *Journal of Neuroscience*.

[21] Patrushev M., Kasymov V., Patrusheva V., Ushakova T., Gogvadze V., Gaziev A. (2004). Mitochondrial permeability transition triggers the release of mtDNA fragments. *Cellular and Molecular Life Sciences*.

[22] Conos S. A., Chen K. W., De Nardo D. (2017). Active MLKL triggers the NLRP3 inflammasome in a cell-intrinsic manner. *Proceedings of the National Academy of Sciences*.

[23] Datta S., Cano M., Ebrahimi K., Wang L., Handa J. T. (2017). The impact of oxidative stress and inflammation on RPE degeneration in non-neovascular AMD. *Progress in Retinal and Eye Research*.

[24] Tokarz P., Kaarniranta K., Blasiak J. (2013). Role of antioxidant enzymes and small molecular weight antioxidants in the pathogenesis of age-related macular degeneration (AMD). *Biogerontology*.

[25] Balak D. M. W., van Doorn M. B., Arbeit R. D. (2017). IMO-8400, a toll-like receptor 7, 8, and 9 antagonist, demonstrates clinical activity in a phase 2a, randomized, placebo-controlled trial in patients with moderate-to-severe plaque psoriasis. *Clinical Immunology*.

[26] Langmann T. (2007). Microglia activation in retinal degeneration. *Journal of Leukocyte Biology*.

[27] Madeira M. H., Rashid K., Ambrósio A. F., Santiago A. R., Langmann T. (2018). Blockade of microglial adenosine A2A receptor impacts inflammatory mechanisms, reduces ARPE-19 cell dysfunction and prevents photoreceptor loss in vitro. *Scientific Reports*.

[28] Lakkaraju A., Toops K. A., Xu J. (2014). Should I stay or should I go? Trafficking of sub-lytic MAC in the retinal pigment epithelium. *Advances in Experimental Medicine and Biology*.

[29] Natoli R., Fernando N., Madigan M. (2017). Microglia-derived IL-1*β* promotes chemokine expression by Müller cells and RPE in focal retinal degeneration. *Molecular Neurodegeneration*.

[30] Parisi L., Gini E., Baci D. (2018). Macrophage polarization in chronic inflammatory diseases: killers or builders?. *Journal of immunology research*.

[31] Finger J. N., Lich J. D., Dare L. C. (2012). Autolytic proteolysis within the function to find domain (FIIND) is required for NLRP1 inflammasome activity. *Journal of Biological Chemistry*.

[32] Bhutto I. A., McLeod D. S., Jing T., Sunness J. S., Seddon J. M., Lutty G. A. (2016). Increased choroidal mast cells and their degranulation in age-related macular degeneration. *British Journal of Ophthalmology*.

[33] Malsy J., Alvarado A. C., Lamontagne J. O., Strittmatter K., Marneros A. G. (2020). Distinct effects of complement and of NLRP3- and non-NLRP3 inflammasomes for choroidal neovascularization. *Elife*.

[34] Braig D., Nero T. L., Koch H.-G. (2017). Transitional changes in the CRP structure lead to the exposure of proinflammatory binding sites. *Nature Communications*.

[35] Kerr J. F., Wyllie A. H., Currie A. R. (1972). Apoptosis: a basic biological phenomenon with wide-ranging implications in tissue kinetics. *British Journal of Cancer*.

[36] Korsmeyer S. J., Wei M. C., Saito M., Weiler S., Oh K. J., Schlesinger P. H. (2000). Pro-apoptotic cascade activates BID, which oligomerizes BAK or BAX into pores that result in the release of cytochrome c. *Cell Death and Differentiation*.

[37] Fritsche L. G., Fariss R. N., Stambolian D., Abecasis G. R., Curcio C. A., Swaroop A. (2014). Age-related macular degeneration: genetics and biology coming together. *Annual Review of Genomics and Human Genetics*.

[38] Münch C. (2018). The different axes of the mammalian mitochondrial unfolded protein response. *BMC Biology*.

[39] Gray D. C., Mahrus S., Wells J. A. (2010). Activation of specific apoptotic caspases with an engineered small-molecule-activated protease. *Cell*.

[40] Flamm J., Hartung S., Gänger S., Maigler F., Pitzer C., Schindowski K. (2021). Establishment of an olfactory region-specific intranasal delivery technique in mice to target the central nervous system. *Frontiers in Pharmacology*.

[41] Susin S. A., Lorenzo H. K., Zamzami N. (1999). Mitochondrial release of caspase-2 and-9 during the apoptotic process. *Journal of Experimental Medicine*.

[42] Devarajan G., Niven J., Forrester J. V., Crane I. J. (2016). Retinal pigment epithelial cell apoptosis is influenced by a combination of macrophages and soluble mediators present in age-related macular degeneration. *Current Eye Research*.

[43] Ouyang X., Yang J., Hong Z., Wu Y., Xie Y., Wang G. (2020). Mechanisms of blue light-induced eye hazard and protective measures: a review. *Biomedicine and Pharmacotherapy*.

[44] Rinninella E., Raoul P., Cintoni M. (2019). What is the healthy gut microbiota composition? A changing ecosystem across age, environment, diet, and diseases. *Microorganisms*.

[45] Shaban L., Chen Y., Fasciano A. C. (2018). A 3D intestinal tissue model supports Clostridioides difficile germination, colonization, toxin production and epithelial damage. *Anaerobe*.

[46] Ahlawat S., Asha, Sharma K. K. (2021). Gut-organ axis: a microbial outreach and networking. *Letters in Applied Microbiology*.

[47] Schoeler M., Caesar R. (2019). Dietary lipids, gut microbiota and lipid metabolism. *Reviews in Endocrine and Metabolic Disorders*.

[48] Jaul E., Barron J. (2017). Age-related diseases and clinical and public health implications for the 85 Years old and over population. *Frontiers in Public Health*.

[49] Rowan S., Jiang S., Korem T. (2017). Involvement of a gut-retina axis in protection against dietary glycemia-induced age-related macular degeneration. *Proceedings of the National Academy of Sciences*.

[50] Rowan S., Taylor A. (2018). Gut microbiota modify risk for dietary glycemia-induced age-related macular degeneration. *Gut Microbes*.

[51] Yissachar N., Zhou Y., Ung L. (2017). An intestinal organ culture system uncovers a role for the nervous system in microbe-immune crosstalk. *Cell*.

[52] Zysset-Burri D. C., Keller I., Berger L. E. (2020). Associations of the intestinal microbiome with the complement system in neovascular age-related macular degeneration. *NPJ Genomic Medicine*.

[53] van Leeuwen E. M., Emri E., Merle B. M. (2018). A new perspective on lipid research in age-related macular degeneration. *Progress in Retinal and Eye Research*.

[54] Rozing M. P., Durhuus J. A., Krogh Nielsen M. (2020). Age-related macular degeneration: a two-level model hypothesis. *Progress in Retinal and Eye Research*.

[55] Curcio C. A., Johnson M., Rudolf M., Huang J. D. (2011). The oil spill in ageing Bruch membrane. *British Journal of Ophthalmology*.

[56] Talbot C. P. J., Plat J., Ritsch A., Mensink R. P. (2018). Determinants of cholesterol efflux capacity in humans. *Progress in Lipid Research*.

[57] Albert A., Alexander D., Boesze-Battaglia K. (2016). Cholesterol in the rod outer segment: a complex role in a simple system. *Chemistry and Physics of Lipids*.

[58] Acar İ. E., Lores-Motta L., Colijn J. M. (2020). Integrating metabolomics, genomics, and disease pathways in age-related macular degeneration: the EYE-RISK consortium. *Ophthalmology*.

[59] Olofsson S., Borèn J. (2005). Apolipoprotein B: a clinically important apolipoprotein which assembles atherogenic lipoproteins and promotes the development of atherosclerosis. *Journal of Internal Medicine*.

[60] Kelly U. L., Grigsby D., Cady M. A. (2020). High-density lipoproteins are a potential therapeutic target for age-related macular degeneration. *Journal of Biological Chemistry*.

[61] Vavvas D. G., Daniels A. B., Kapsala Z. G. (2016). Regression of some high-risk features of age-related macular degeneration (AMD) in patients receiving intensive statin treatment. *EBioMedicine*.

[62] Zheng W., Mast N., Saadane A., Pikuleva I. A. (2015). Pathways of cholesterol homeostasis in mouse retina responsive to dietary and pharmacologic treatments. *Journal of Lipid Research*.

[63] Jobling A. I., Guymer R. H., Vessey K. A. (2015). Nanosecond laser therapy reverses pathologic and molecular changes in age-related macular degeneration without retinal damage. *The FASEB Journal*.

[64] Dikic I., Elazar Z. (2018). Mechanism and medical implications of mammalian autophagy. *Nature Reviews Molecular Cell Biology*.

[65] Galluzzi L., Baehrecke E. H., Ballabio A. (2017). Molecular definitions of autophagy and related processes. *The EMBO Journal*.

[66] Kaarniranta K., Uusitalo H., Blasiak J. (2020). Mechanisms of mitochondrial dysfunction and their impact on age-related macular degeneration. *Progress in Retinal and Eye Research*.

[67] Saadat K. A. S. M., Murakami Y., Tan X. (2014). Inhibition of autophagy induces retinal pigment epithelial cell damage by the lipofuscin fluorophore A2E. *FEBS Open Bio*.

[68] Felszeghy S., Viiri J., Paterno J. J. (2019). Loss of NRF-2 and PGC-1*α* genes leads to retinal pigment epithelium damage resembling dry age-related macular degeneration. *Redox Biology*.

[69] Golestaneh N., Chu Y., Xiao Y.-Y., Stoleru G. L., Theos A. C. (2017). Dysfunctional autophagy in RPE, a contributing factor in age-related macular degeneration. *Cell Death and Disease*.

[70] Kaarniranta K., Salminen A., Eskelinen E. L., Kopitz J. (2009). Heat shock proteins as gatekeepers of proteolytic pathways-Implications for age-related macular degeneration (AMD). *Ageing Research Reviews*.

[71] Keeling E., Lotery A. J., Tumbarello D. A., Ratnayaka J. A. (2018). Impaired cargo clearance in the retinal pigment epithelium (RPE) underlies irreversible blinding diseases. *Cells*.

[72] Liu L., Zhao X., Wang Q. (2016). Prosteatotic and protective components in a unique model of fatty liver: gut microbiota and suppressed complement system. *Scientific Reports*.

[73] Osman R. H., Shao D., Liu L. (2016). Expression of mitochondria-related genes is elevated in overfeeding-induced goose fatty liver. *Comparative Biochemistry and Physiology Part B: Biochemistry and Molecular Biology*.

[74] Wu L., Tan X., Liang L. (2017). The role of mitochondria-associated reactive oxygen species in the amyloid *β* induced production of angiogenic factors b y ARPE-19 cells. *Current Molecular Medicine*.

[75] Kunchithapautham K., Atkinson C., Rohrer B. (2014). Smoke exposure causes endoplasmic reticulum stress and lipid accumulation in retinal pigment epithelium through oxidative stress and complement activation. *Journal of Biological Chemistry*.

[76] Mazkereth N., Rocca F., Schubert J. R. (2016). Complement triggers relocation of Mortalin/GRP75 from mitochondria to the plasma membrane. *Immunobiology*.

[77] Tan S. M., Ziemann M., Thallas-Bonke V. (2020). Complement C5a induces renal injury in diabetic kidney disease by disrupting mitochondrial metabolic agility. *Diabetes*.

[78] Ishii M., Beeson G., Beeson C., Rohrer B. (2021). Mitochondrial C3a receptor activation in oxidatively stressed epithelial cells reduces mitochondrial respiration and metabolism. *Frontiers in Immunology*.

[79] Lohman R.-J., Hamidon J. K., Reid R. C. (2017). Exploiting a novel conformational switch to control innate immunity mediated by complement protein C3a. *Nature Communications*.

[80] Tan L. X., Toops K. A., Lakkaraju A. (2016). Protective responses to sublytic complement in the retinal pigment epithelium. *Proceedings of the National Academy of Sciences*.

[81] Morgan M. J., Liu Z.-G. (2011). Crosstalk of reactive oxygen species and NF-*κ*B signaling. *Cell Research*.

[82] Lipitsä T., Naukkarinen A., Laitala J., Harvima I. T. (2016). Complement C3 is expressed by mast cells in cutaneous vasculitis and is degraded by chymase. *Archives of Dermatological Research*.

[83] Arbore G., Kemper C. (2016). A novel complement-metabolism-inflammasome axis as a key regulator of immune cell effector function. *European Journal of Immunology*.

[84] Chen W., Zhao M., Zhao S. (2017). Activation of the TXNIP/NLRP3 inflammasome pathway contributes to inflammation in diabetic retinopathy: a novel inhibitory effect of minocycline. *Inflammation Research*.

[85] Rawal N., Pangburn M. K. (1998). C5 convertase of the alternative pathway of complement. *Journal of Biological Chemistry*.

[86] Bhan C., Dipankar P., Chakraborty P., Sarangi P. P. (2016). Role of cellular events in the pathophysiology of sepsis. *Inflammation Research*.

[87] Armento A., Schmidt T. L., Sonntag I. (2021). CFH loss in human RPE cells leads to inflammation and complement system dysregulation via the NF-*κ*B pathway. *International Journal of Molecular Sciences*.

[88] Medjeral-Thomas N. R., Pickering M. C.-B., Lomax-Browne H. J. (2017). Circulating complement factor H-related proteins 1 and 5 correlate with disease activity in IgA nephropathy. *Kidney International*.

[89] Liszewski M. K., Kolev M., Le Friec G. (2013). Intracellular complement activation sustains T cell homeostasis and mediates effector differentiation. *Immunity*.

[90] Ma W., Zhao L., Fontainhas A. M., Fariss R. N., Wong W. T. (2009). Microglia in the mouse retina alter the structure and function of retinal pigmented epithelial cells: a potential cellular interaction relevant to AMD. *PLoS One*.

[91] Kim M.-O., Moon D.-O., Jung J. M., Lee W. S., Choi Y. H., Kim G. Y. (2011). Agaricus blazei extract induces apoptosis through ROS-dependent JNK activation involving the mitochondrial pathway and suppression of constitutive NF-*κ*B in THP-1 cells. *Evidence-based Complementary and Alternative Medicine*.

[92] Ganbarjeddi S., Azimi A., Zadi Heydarabad M. (2020). Apoptosis induced by prednisolone occurs without altering the promoter methylation of BAX and BCL-2 genes in acute lymphoblastic leukemia cells CCRF-CEM. *Asian Pacific Journal of Cancer Prevention*.

[93] Kurihara R., Yamaoka K., Sawamukai N. (2010). C5a promotes migration, proliferation, and vessel formation in endothelial cells. *Inflammation Research*.

[94] Monk P. N., Scola A., Madala P., Fairlie D. P. (2007). Function, structure and therapeutic potential of complement C5a receptors. *British Journal of Pharmacology*.

[95] Hou J., Yang Y., Zhang T., Zhu C., Lv K. (2021). The effects of P53 in the globular heads of the C1q receptor in gastric carcinoma cell apoptosis are exerted via a mitochondrial-dependent pathway. *Doklady Biochemistry and Biophysics*.

[96] Tropini C., Earle K. A., Huang K. C., Sonnenburg J. L. (2017). The gut microbiome: connecting spatial organization to function. *Cell Host and Microbe*.

[97] Qi H., Wei J., Gao Y. (2020). Reg4 and complement factor D prevent the overgrowth of E. coli in the mouse gut. *Communications Biology*.

[98] Choi Y. J., Kim J. E., Lee S. J. (2021). Dysbiosis of fecal microbiota from complement 3 knockout mice with constipation phenotypes contributes to development of defecation delay. *Frontiers in Physiology*.

[99] Carabotti M., Scirocco A., Maselli M. A., Severi C. (2015). The gut-brain axis: interactions between enteric microbiota, central and enteric nervous systems. *Annals of Gastroenterology*.

[100] Lintner K. E., Patwardhan A., Rider L. G. (2016). Gene copy-number variations (CNVs) of complement C4 and C4A deficiency in genetic risk and pathogenesis of juvenile dermatomyositis. *Annals of the Rheumatic Diseases*.

[101] Floyd J. L., Grant M. B. (2020). The gut-eye Axis: lessons learned from murine models. *Ophthalmology and Therapy*.

[102] Landowski M., Kelly U., Klingeborn M. (2019). Human complement factor H Y402H polymorphism causes an age-related macular degeneration phenotype and lipoprotein dysregulation in mice. *Proceedings of the National Academy of Sciences*.

[103] Tsuru H., Osaka M., Hiraoka Y., Yoshida M. (2020). HFD-induced hepatic lipid accumulation and inflammation are decreased in Factor D deficient mouse. *Scientific Reports*.

[104] Yin C., Ackermann S., Ma Z. (2019). ApoE attenuates unresolvable inflammation by complex formation with activated C1q. *Nature Medicine*.

[105] Hyttinen J. M. T., Viiri J., Kaarniranta K., Błasiak J. (2018). Mitochondrial quality control in AMD: does mitophagy play a pivotal role?. *Cellular and Molecular Life Sciences*.

[106] Clark S. J., Bishop P. N. (2018). The eye as a complement dysregulation hotspot. *Seminars in Immunopathology*.

[107] Nguyen H., Kuril S., Bastian D. (2018). Complement C3a and C5a receptors promote GVHD by suppressing mitophagy in recipient dendritic cells. *Journal of Clinical Investigation Insight*.

[108] Lv Q., Yang F., Chen K., Zhang Y. (2016). Autophagy protects podocytes from sublytic complement induced injury. *Experimental Cell Research*.

[109] Yamamoto H., Fara A. F., Dasgupta P., Kemper C. (2013). CD46: the “multitasker” of complement proteins. *The International Journal of Biochemistry and Cell Biology*.

[110] Helmy K. Y., Katschke K. J., Gorgani N. N. (2006). CRIg: a macrophage complement receptor required for phagocytosis of circulating pathogens. *Cell*.

[111] Hakobyan S., Harris C. L., Tortajada A. (2008). Measurement of factor H variants in plasma using variant-specific monoclonal antibodies: application to assessing risk of age-related macular degeneration. *Investigative Opthalmology and Visual Science*.

[112] Cipriani V., Tierney A., Griffiths J. R. (2021). Beyond factor H: the impact of genetic-risk variants for age-related macular degeneration on circulating factor-H-like 1 and factor-H-related protein concentrations. *The American Journal of Human Genetics*.

[113] Borras C., Canonica J., Jorieux S. (2019). CFH exerts anti-oxidant effects on retinal pigment epithelial cells independently from protecting against membrane attack complex. *Scientific Reports*.

[114] Józsi M., Schneider A. E., Kárpáti É., Sándor N. (2019). Complement factor H family proteins in their non-canonical role as modulators of cellular functions. *Seminars in Cell and Developmental Biology*.

[115] Klein R. J., Zeiss C., Chew E. Y. (2005). Complement factor H polymorphism in age-related macular degeneration. *Science*.

[116] Cipriani V., Lorés-Motta L., He F. (2020). Increased circulating levels of Factor H-Related Protein 4 are strongly associated with age-related macular degeneration. *Nature Communications*.

[117] Hageman G. S., Hancox L. S., Taiber A. J. (2006). Extended haplotypes in the complement factor H (CFH) and CFH-related (CFHR) family of genes protect against age-related macular degeneration: characterization, ethnic distribution and evolutionary implications. *Annals of Medicine*.

[118] Sodi A., Passerini I., Bacherini D. (2018). CFH Y402H polymorphism in Italian patients with age-related macular degeneration, retinitis pigmentosa, and Stargardt disease. *Ophthalmic Genetics*.

[119] Han X., Gharahkhani P., Mitchell P., Liew G., Hewitt A. W., MacGregor S. (2020). Genome-wide meta-analysis identifies novel loci associated with age-related macular degeneration. *Journal of Human Genetics*.

[120] Maugeri A., Barchitta M., Agodi A. (2019). The association between complement factor H rs1061170 polymorphism and age-related macular degeneration: a comprehensive meta-analysis stratified by stage of disease and ethnicity. *Acta Ophthalmologica*.

[121] Zouache M. A., Bennion A., Hageman J. L., Pappas C., Richards B. T., Hageman G. S. (2020). Macular retinal thickness differs markedly in age-related macular degeneration driven by risk polymorphisms on chromosomes 1 and 10. *Scientific Reports*.

[122] Maller J., George S., Purcell S. (2006). Common variation in three genes, including a noncoding variant in CFH, strongly influences risk of age-related macular degeneration. *Nature Genetics*.

[123] Clark S. J., Bishop P. N. (2014). Role of factor H and related proteins in regulating complement activation in the macula, and relevance to age-related macular degeneration. *Journal of Clinical Medicine*.

[124] Heesterbeek T. J., Lechanteur Y. T. E., Lorés-Motta L. (2020). Complement activation levels are related to disease stage in AMD. *Investigative Opthalmology and Visual Science*.

[125] Armento A., Ueffing M., Clark S. J. (2021). The complement system in age-related macular degeneration. *Cellular and Molecular Life Sciences*.

[126] Fernandez-Godino R., Pierce E. A. (2018). C3a triggers formation of sub-retinal pigment epithelium deposits via the ubiquitin proteasome pathway. *Scientific Reports*.

[127] Yu M., Zou W., Peachey N. S., McIntyre T. M., Liu J. (2012). A novel role of complement in retinal degeneration. *Investigative Opthalmology and Visual Science*.

[128] Rohrer B., Frazer-Abel A., Leonard A. (2019). Association of age-related macular degeneration with complement activation products, smoking, and single nucleotide polymorphisms in South Carolinians of European and African descent. *Molecular Vision*.

[129] Propson N. E., Roy E. R., Litvinchuk A., Köhl J., Zheng H. (2021). Endothelial C3a receptor mediates vascular inflammation and blood-brain barrier permeability during aging. *The Journal of Clinical Investigation*.

[130] Lechner J., Chen M., Hogg R. E. (2016). Higher plasma levels of complement C3a, C4a and C5a increase the risk of subretinal fibrosis in neovascular age-related macular degeneration: complement activation in AMD. *Immunity and Ageing*.

[131] Lu F., Liu S., Hao Q. (2018). Association between complement factor C2/C3/CFB/CFH polymorphisms and age-related macular degeneration: a meta-analysis. *Genetic Testing and Molecular Biomarkers*.

[132] Zhang J., Li S., Hu S., Yu J., Xiang Y. (2018). Association between genetic variation of complement C3 and the susceptibility to advanced age-related macular degeneration: a meta-analysis. *BMC Ophthalmology*.

[133] Yates J. R. W., Sepp T., Matharu B. K. (2007). Complement C3 variant and the risk of age-related macular degeneration. *New England Journal of Medicine*.

[134] Grassmann F., Cantsilieris S., Schulz-Kuhnt A. S. (2016). Multiallelic copy number variation in the complement component 4A (C4A) gene is associated with late-stage age-related macular degeneration (AMD). *Journal of Neuroinflammation*.

[135] Cobos E., Recalde S., Anter J. (2018). Association between CFH, CFB, ARMS2, SERPINF1, VEGFR1 and VEGF polymorphisms and anatomical and functional response to ranibizumab treatment in neovascular age-related macular degeneration. *Acta Ophthalmologica*.

[136] Seddon J. M., George S., Rosner B., Rifai N. (2005). Progression of age-related macular degeneration: prospective assessment of C-reactive protein, interleukin 6, and other cardiovascular biomarkers. *Archives of Ophthalmology*.

[137] Altay L., Sitnilska V., Schick T. (2019). Early local activation of complement in aqueous humour of patients with age-related macular degeneration. *Eye*.

[138] Lakkaraju A., Umapathy A., Tan L. X. (2020). The cell biology of the retinal pigment epithelium. *Progress in Retinal and Eye Research*.

[139] Pilotti C., Greenwood J., Moss S. E. (2020). Functional evaluation of AMD-associated risk variants of complement factor B. *Investigative Opthalmology and Visual Science*.

[140] Sun C., Zhao M., Li X. (2012). CFB/C2 gene polymorphisms and risk of age-related macular degeneration: a systematic review and meta-analysis. *Current Eye Research*.

[141] Wu J., Sun X. (2019). Complement system and age-related macular degeneration: drugs and challenges. *Drug Design, Development and Therapy*.

[142] Wu X., Hutson I., Akk A. M. (2018). Contribution of adipose-derived factor D/adipsin to complement alternative pathway activation: lessons from lipodystrophy. *The Journal of Immunology*.

[143] Barratt J., Weitz I. (2021). Complement factor D as a strategic target for regulating the alternative complement pathway. *Frontiers in Immunology*.

[144] Łukawska E., Polcyn-Adamczak M., Niemir Z. I. (2018). The role of the alternative pathway of complement activation in glomerular diseases. *Clinical and Experimental Medicine*.

[145] Dobó J., Szakács D., Oroszlán G. (2016). MASP-3 is the exclusive pro-factor D activator in resting blood: the lectin and the alternative complement pathways are fundamentally linked. *Scientific Reports*.

[146] Yaspan B. L., Williams D. F., Holz F. G. (2017). Targeting factor D of the alternative complement pathway reduces geographic atrophy progression secondary to age-related macular degeneration. *Science Translational Medicine*.

[147] Rohrer B., Guo Y., Kunchithapautham K., Gilkeson G. S. (2007). Eliminating complement factor D reduces photoreceptor susceptibility to light-induced damage. *Investigative Opthalmology and Visual Science*.

[148] Tian Y., Kijlstra A., Webers C. A. B., Berendschot T. T. (2015). Lutein and Factor D: two intriguing players in the field of age-related macular degeneration. *Archives of Biochemistry and Biophysics*.

[149] Zeng J., Chen Y., Tong Z. (2010). Lack of association of CFD polymorphisms with advanced age-related macular degeneration. *Molecular Vision*.

[150] Java A., Pozzi N., Love-Gregory L. D. (2019). A multimodality approach to assessing factor I genetic variants in atypical hemolytic uremic syndrome. *Kidney international reports*.

[151] Lachmann P. J. (2019). The story of complement factor I. *Immunobiology*.

[152] Lashkari K., Teague G., Chen H. (2018). A monoclonal antibody targeting amyloid *β* (A*β*) restores complement factor I bioactivity: potential implications in age-related macular degeneration and Alzheimer’s disease. *PLoS One*.

[153] de Jong S., Volokhina E. B., de Breuk A. (2020). Effect of rare coding variants in the CFI gene on Factor I expression levels. *Human Molecular Genetics*.

[154] Hallam T. M., Marchbank K. J., Harris C. L. (2020). Rare genetic variants in complement factor I lead to low FI plasma levels resulting in increased risk of age-related macular degeneration. *Investigative Opthalmology and Visual Science*.

[155] Fernandez-Godino R., Bujakowska K. M., Pierce E. A. (2018). Changes in extracellular matrix cause RPE cells to make basal deposits and activate the alternative complement pathway. *Human Molecular Genetics*.

[156] Khan A. H., Sutton J., Cree A. J. (2021). Prevalence and phenotype associations of complement factor I mutations in geographic atrophy. *Human Mutation*.

[157] Tan P. L., Garrett M. E., Willer J. R. (2017). Systematic functional testing of rare variants: contributions of CFI to age-related macular degeneration. *Investigative Opthalmology and Visual Science*.

[158] Lay E., Nutland S., Smith J. E. (2015). Complotype affects the extent of down-regulation by Factor I of the C3b feedback cycle in vitro. *Clinical and Experimental Immunology*.

[159] de Jong S., Gagliardi G., Garanto A. (2021). Implications of genetic variation in the complement system in age-related macular degeneration. *Progress in Retinal and Eye Research*.

[160] Mastaglio S., Ruggeri A., Risitano A. M. (2020). The first case of COVID-19 treated with the complement C3 inhibitor AMY-101. *Clinical immunology*.

[161] Qin S., Dong N., Yang M., Wang J., Feng X., Wang Y. (2021). Complement inhibitors in age-related macular degeneration: a potential therapeutic option. *Journal of immunology research*.

[162] Maugeri A., Barchitta M., Mazzone M. G., Giuliano F., Agodi A. (2018). Complement system and age-related macular degeneration: implications of gene-environment interaction for preventive and personalized medicine. *BioMed Research International*.

